# Assumptions in ecosystem service assessments: Increasing transparency for conservation

**DOI:** 10.1007/s13280-020-01379-9

**Published:** 2020-09-11

**Authors:** Matthias Schröter, Emilie Crouzat, Lisanne Hölting, Julian Massenberg, Julian Rode, Mario Hanisch, Nadja Kabisch, Julia Palliwoda, Jörg A. Priess, Ralf Seppelt, Michael Beckmann

**Affiliations:** 1 UFZ - Helmholtz Centre for Environmental Research, Permoserstr. 15, 04318 Leipzig, Germany; 2 Université Grenoble Alpes, INRAE, LESSEM, 2 Rue de la Papeterie, 38402 Saint-Martin-d’Hères, France; 3 German Centre for Integrative Biodiversity Research (iDiv) Halle-Jena-Leipzig, Deutscher Platz 5e, 04103 Leipzig, Germany; 4 Department of Geography, Humboldt-Universität zu Berlin, Unter den Linden 6, 10099 Berlin, Germany; 5 Institute of Geoscience & Geography, Martin-Luther University Halle-Wittenberg, 06099 Halle, Germany

**Keywords:** Assessment, Decision-making, Ecosystem services, Environmental ethics, Mapping, Valuation

## Abstract

Conservation efforts are increasingly supported by ecosystem service assessments. These assessments depend on complex multi-disciplinary methods, and rely on a number of assumptions which reduce complexity. If assumptions are ambiguous or inadequate, misconceptions and misinterpretations may arise when interpreting results of assessments. An interdisciplinary understanding of assumptions in ecosystem service science is needed to provide consistent conservation recommendations. Here, we synthesise and elaborate on 12 prevalent types of assumptions in ecosystem service assessments. These comprise conceptual and ethical foundations of the ecosystem service concept, assumptions on data collection, indication, mapping, and modelling, on socio-economic valuation and value aggregation, as well as about using assessment results for decision-making. We recommend future assessments to increase transparency about assumptions, and to test and validate them and their potential consequences on assessment reliability. This will support the taking up of assessment results in conservation science, policy and practice.

**Electronic supplementary material:**

The online version of this article (10.1007/s13280-020-01379-9) contains supplementary material, which is available to authorized users.

## What are assumptions in ecosystem service assessments?

Ecosystem services (ES) have become an important concept to justify and underpin conservation efforts. ES have been acknowledged as a crucial conservation framework, with applications for protected areas and management of used landscapes (e.g. Kremen and Merenlender 2018). ES assessments, i.e. measurements, quantifications or valuations of ES, have seen remarkable methodological advances in recent years and frequently employ multi-, inter- and transdisciplinary methods that rely on a series of assumptions. While assumptions are made in many fields of research, the interdisciplinary nature of ES assessments leads to assumptions being made on different levels: from philosophical reasoning and normative preconceptions to disciplinary methods. Moreover, assumptions are discipline-specific and are based on diverse research paradigms and their philosophical foundations. Thereby, it becomes challenging to account for all assumptions made in an ES assessment. While methodological debates on the appropriateness of assumptions have taken place within disciplines (e.g. economics, Wegner and Pascual 2011), an overview of the variety of assumptions made in ES assessments as well as their potential consequences for conservation is lacking.

Assumptions are defined here as implicit or explicit statements that are postulated to be true or taken for granted without providing evidence or proof of their validity (adapted from Cambridge Dictionary 2019). Assumptions serve to handle and reduce the complexity of interactions in social–ecological systems (Evans 2019) and are necessary for scientific work. However, when they are not made explicit, or are ambiguous, one-sided or inadequate, assumptions can lead to misconceptions and misinterpretations. Therefore, they can reduce the usefulness of ES assessments, especially when operationalising conservation applications. It remains challenging to achieve an in-depth understanding of the extent to which assumptions are influencing the results of ES assessments, potentially biassing the results and thereby conservation recommendations. ES assessments are often used to support conservation decisions and can involve normative or prescriptive implications (e.g. as part of cost–benefit analyses for or against infrastructure development). Therefore, awareness and reflection of assumptions are crucial to better evaluate the adequacy of ES assessments to justify the conservation of nature.

In order to increase interdisciplinary awareness on such assumptions and to strengthen the application of ES assessments, we here synthesise types of assumptions that are commonly made in ES assessments. We develop an overview on and typology of assumptions and explain them to an interdisciplinary audience, while neither claiming that list being exhaustive nor intending to assess the prevalence of these assumptions. We furthermore illustrate the conservation relevance of each of these assumption types. Finally, we discuss opportunities to improve transparency on assumptions, and how conservation scientists, practitioners and decision-makers can better handle assumptions to establish a good practice.

## Methods

A typology of assumptions in ES assessments was developed based on expert knowledge during 2 half-day expert workshops by 14 participants (11 of which are authors of this paper). Experts represented multiple, often cross-cutting disciplines (environmental sciences [6], environmental and ecological economics [2], urban ecology [2], terrestrial/geo-ecology [2], behavioural economics [1], conservation biology [1]) and different career stages (see Table S1 in Supplementary Material). Each participant identified five assumptions that they judged most relevant in ES assessments according to their experience. Similar assumptions were then grouped into categories through an open discussion. A preliminary list of assumptions was built as a collaborative team effort. This list was confirmed and adapted through a systematic literature review. We searched the Web of Science on 12 June 2018 for TITLE: [“ecosystem service*” AND (assess* OR map* OR account* OR valu* OR quantif* OR analy* OR indic*)]. Conceptual or review papers were excluded. To focus on frequently cited papers that indicate common practice, we restricted the search to the ten most cited papers published between 2010 and 2016. Our study does not include papers published after 2016 as younger papers did not have enough citations.

**Fig. 1:**
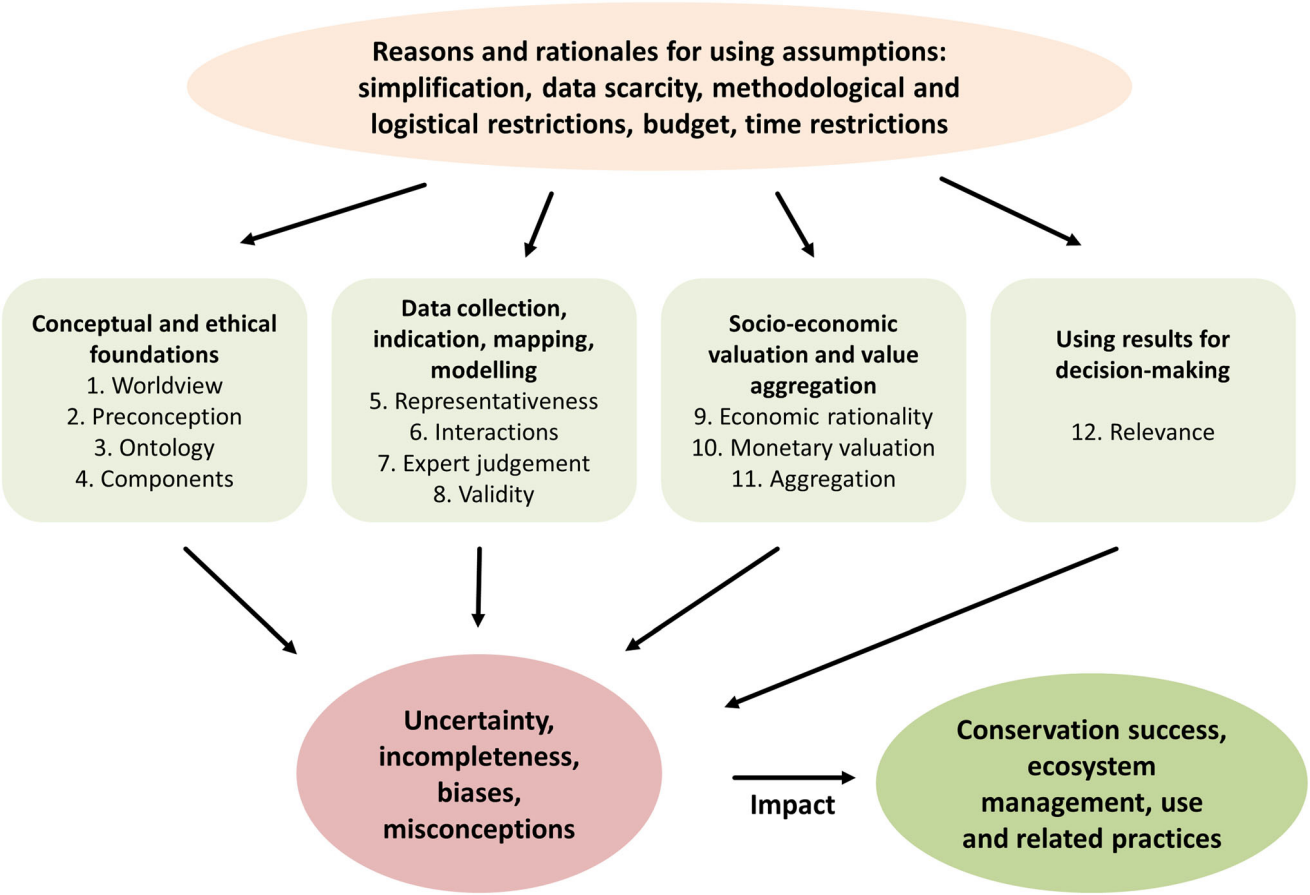
Twelve types of assumptions in four domains contribute to uncertainties, incompleteness, biases or misconceptions of ecosystem service assessments. These, in turn, can have impacts on the success of conservation decisions and ecosystem management and use informed by these assessments

All 70 papers (Table S3 in Supplementary Material) were distributed among the authors, read in depth and checked against the preliminary list of assumptions by using a standardised data extraction sheet (Table S2 in Supplementary Material). While no new types of assumptions were added during the review, we focused on synthesising types of assumptions as far as possible, aiming for broad types applicable across ES assessments. This list was then clustered by the authors into four domains to define generic types of assumptions. In the second workshop, the final list of assumptions and related categories were discussed and agreed upon by all participants. Assumption 3 (for numbering please refer to Fig. 1 and Table 1) was added based on relevant comments during peer-review.

## What types of assumptions are common in ES assessments?

We structure assumptions into four domains: Conceptual and ethical foundations (“Conceptual and ethical foundations of the ES concept” section); Data collection, indication, mapping, and modelling (“Data collection, indication, mapping, and modelling” section); Socio-economic valuation and value aggregation (“Socio-economic valuation and value aggregation” section); and Using results for decision-making (“Using results for decision-making” section). The following sections provide detailed descriptions for each of the 12 assumptions and their implications for conservation (see also Fig. 1; Table 1 for summaries).

**Table 1:** Conservation relevance of assumptions and selected strategies to deal with assumptions

Assumption	Conservation relevance	Strategies to deal with assumptions for conservation science	Strategies to deal with assumptions for conservation policy and practice
(1) *Worldview* implicit normative preconceptions of the human–nature relationship	– Potentially neglecting importance of other arguments for conservation (intrinsic, relational)	– Address different values in ES assessments – Carry out assessments for biodiversity and ES separately	– Acknowledge the incompleteness of ES assessments with respect to different worldviews
(2) *Preconception* ecosystems being good per se	– Service-providing species might cause disservices (invasive species/large predators)	– Assess negative contributions/disservices next to services	– Acknowledge value conflicts when managing populations of service/disservice-providing species
(3) *Ontology* appropriate use of the terms “ecosystem” and “services”	– People connect to “nature”, not to “ecosystems” – Strict wording might cause rejections among stakeholders	– Adopt the conceptual language to specific contexts – Apply context-specific perspectives for place-based assessments	– Make use of local knowledge – When communicating to stakeholders adopt the language and step away from conceptual-scientific considerations
(4) *Components* ES components used interchangeably	– Misinterpretations from ES assessments, e.g. no actual use, but only potential provision in access-restricted areas – Hiding potential overuse when actual use is higher than sustainable capacity	– Explicitly name assessed components – Reduce conceptual fuzziness through generally accepted framework and shared common language, e.g. standardised essential variables of ES	– Consider actual use of ES, as well as potential (sustainable) provision when managing protected areas
(5) *Representativeness* secondary data, time, space are representative	– Credibility and robustness limited when transferring ES data from different context to e.g. a protected area	– Ask local community about their knowledge for context-specific place-based assessments – Use adjusted value transfers and meta-analytic value functions – Collect field data to evaluate uncertainties in transferred data	– Reconsider the applicability of transferred data from outside protected areas, in particular with respect to management and access restrictions – Locally adjusted values might more strongly stress the necessity for conservation
(6) *Interactions* ES are independent entities	– High multifunctionality does not tell about conservation values, e.g. rare ecosystems are not the most multifunctional – Trade-offs between provisioning and regulating services, including provision of habitat, and biodiversity and between provisioning and cultural services, such as recreational use of cultural landscapes of high conservation value	– Assess overuse and negative indirect effects related to joint use of ES (e.g. agriculture, forestry) – Study interactions over time and across space – Consider functional mechanisms relating ES and biodiversity (e.g. functional trait-based models)	– Consider rarity next to multifunctionality/number of ES – Conservation-compatibility of ES: where do (access) restrictions apply, e.g. for cultural or non-material uses e.g. recreation
(7) *Expert judgement* estimation of ES quantities is appropriate	– Results depend on panel of experts involved, in which conservation aspects or ES may be misjudged or overlooked	– Ensure that conservation experts are involved – Validate with field data, assess and report uncertainties – Test effects of different scoring approaches – Do not consider expert judgements as perfect substitute for empirical evidence and regard that they may change over time	– Consider different types of expertise, including lay expertise, indigenous and local knowledge, technical knowledge – Validate/reconsider prior conservation efforts if empirical data are available
(8) *Validity* ES indicators are credible	– Proxies do not convey the whole picture of an ES and might neglect aspects such as ecological relations	– Build scientific consensus on validity of different indicators – Discuss uncertainties of approximations transparently	– Critically check the conservation relevance of indicators, e.g. their capacity to monitor service-providing species
(9) *Economic rationality* maximising individual utility and well-informed preferences	– Preferences may not be well-established for unfamiliar goods like biodiversity, respondents might not oversee complex consequences for biodiversity	– To ensure well-informed preferences, inform people about ecological complexity underlying ES and include discussions among heterogeneous groups before eliciting values	– Ensure broad inclusion of knowledge on the diversity of values and preferences to avoid undervaluation of ecosystems and biodiversity
(10) *Monetary valuation* approximation of preferences through monetary measures	– Focus on monetary values might exclude other values of biodiversity – Willingness-to-pay is not equal to ability-to-pay for conservation	– Allow for expression of plural values by using various metrics besides monetary measures – Focus not only on monetary value outcomes but also on motives behind preferences	– Consider outcomes of different valuation methods, and be aware that monetary values are only one form of values – Consider relational values that might be of higher conservation relevance
(11) *Aggregation* summing up welfare across individuals, ES and time	– Interpretation of conservation relevance, e.g. the value of a service-providing species over time depends strongly on aggregation rule	– Test robustness of results by using different aggregation procedures – Be explicit about aggregation and discounting choices (e.g. weighting and social discount rates)	– Recognise the diversity of indicators, benefits and values that are attributed to nature and take minority values into account – Consider alternatives to aggregation (multi-criteria approaches)
(12) *Relevance* importance for conservation decisions	– Impact of a stronger integration of ES in conservation policy and practice on classic conservation goals still unclear – Actual uptake unclear	– Ask stakeholders/decision-makers about needs for, e.g. planning or instrument development to inform acceptable uncertainty levels – Analyse post-study uptake of ES assessment results	– Collaborate with scientists on monitoring the actual effect of ES-based management on other conservation goals (e.g. number of species)

### Conceptual and ethical foundations of the ES concept

#### Worldview: ES involve implicit normative preconceptions of the human–nature relationship

Using the ES concept involves implicit normative preconceptions. First, the concept relies on an ethical worldview in which enhancing human well-being is regarded paramount for conservation choices (Davidson 2013). ES represent an instrumental value of nature as a means to enhance human well-being. The mission-oriented framing of many ES studies targets the enhancement of human well-being through the formulation of recommendations for management and policy (Crouzat et al. 2017). Second, the prominently applied cascade model (Haines-Young and Potschin 2010) conceptualises ES as cascading down from biophysical structures to human values. Although progressively refined to account for reciprocal human–nature relationships (Villamagna et al. 2013), ES assessments describe ecosystems as being observer-independent and people as dichotomously distinct entities from nature. Understanding how humans relate to and care for nature might need more integrated metaphors to foster nature conservation (Raymond et al. 2013).

Conceiving the ES concept as supportive argumentation for conservation has been criticised for restricting direct moral obligations to human entities, while humans have indirect moral obligations with respect to nature based on instrumental values (Jax et al. 2013). The underlying framing might neglect other worldviews (such as Mother Earth, Borie and Hulme 2015), more intertwined nature–culture relations, e.g. anchored on local and indigenous worldviews (Díaz et al. 2018), and might undermine other motivations for nature conservation (Rode et al. 2015). However, values that contribute to a good life beyond instrumental values are of conservation relevance and are covered by the ES concept (Schröter et al. 2014). More recent value conceptualisations of human–nature relationships for conservation include a broader set of value dimensions such as intrinsic, relational and social values (e.g. Himes and Muraca 2018).

#### Preconception: Ecosystems are good per se

Another preconception of ES is that nature is considered positive for all humans irrespective of social or cultural background, which can hinder a more differentiated view on positive and negative effects for different groups of people (Díaz et al. 2018). The positive framing of the “services” notion can distract from negative effects of nature on humans, which developed under the notion of “disservices” (Shackleton et al. 2016). A recent review has shown that the consideration of such disservices remains limited in the field (Kadykalo et al. 2019). It is also implicitly assumed that ecosystems are good per se when providing multiple ES which are supposed to maintain long-term ecosystem functioning and provide long-term benefits to the people (Raudsepp-Hearne et al. 2010; Kremen and Merenlender 2018).

A given organism or ecosystem can provide both services and disservices, such as the invasive plants *Acacia* that provide a range of regulating ES such as carbon sequestration or erosion prevention while negatively impacting the perceived natural state of landscapes (Vaz et al. 2017). Also, land abandonment of agricultural lands can be perceived in varied ways, both negatively (e.g. loss of cultural heritage through forest succession) and positively (e.g. increase of scenic beauty) by different groups of people (van der Zanden et al. 2018).

#### Ontology: The terms “ecosystem” and “services” are appropriately used

An ontological assumption (referring to the nature of existence or reality) is how the field conceptualises human–nature relationships with the terms “ecosystem” and “services”. This assumes that both are proper and relevant expressions for the entities they aim to describe. This has been criticised for having drawbacks. Kirchhoff (2019) argues that “ecosystem” is rooted in a natural–scientific, functionalist paradigm and is, in particular in the context of cultural ES, not appropriate to analyse non-material human–nature relationships. People who benefit from these services do not perceive “ecosystems”, but rather “landscapes”, “places”, “wilderness” or “nature”. Moreover, the term “services” might be perceived as rooted in a predominantly economic, functionalist and Western paradigm (e.g. Borie and Hulme 2015; Kirchhoff 2019). “Services” might ontologically not be able to cover all forms of connections that people build with their natural environment for instance with respect to heritage, identity and stewardship (e.g. James 2015).

While terms like “nature” and “service” have their own analytical drawbacks (Kirchhoff 2019), conservation science and practice might benefit from adopting terms that are closer to people’s perception of reality, for instance when communicating multiple benefits or conflicting interests of protected areas. The concept of nature’s contributions to people (Díaz et al. 2018), avoiding both terms “ecosystem” and “services” has arisen not only, but also from such ontological discussions at the global level with decision-makers and stakeholders.

#### Components: ES components are used interchangeably

Ecosystem service assessments can assess the potential supply of ES, and then set it equal to actual use and total demand for ES, following the implicit assumption that services which are provided in one area result in an actual use of the service in that area. According to this assumption, no distinction between different components of ES like ecosystem processes, potential provision, actually used services or demand for services is made (for an overview of interchangeably used terminology see, e.g. Villamagna et al. 2013). An example is the availability of urban green space, which would be an indicator for potential provision of cultural and regulating services, but is used to indicate actual use, e.g. for recreational activities or regulation of water, temperature or air quality (e.g. Derkzen et al. 2015). The rationales behind this assumption are simplification, data scarcity on different aspects of ES (predominantly on ES use and demand), or the need to be pragmatic due to budget and time restrictions.

Demand and actual use might exceed potential provision, resulting in overexploitation of natural capital and pollution. Trade-offs between ES and biodiversity might occur, e.g. through timber harvest (Gonzalez-Redin et al. 2016), fisheries (overfishing) or agricultural production (pesticide run-off, soil compaction or erosion). Differentiating between aspects of ES allows identifying the origin, or determinants, of synergies and trade-offs among ES and biodiversity (Crouzat et al. 2016), a crucial step for enabling conservation management.

### Data collection, indication, mapping, and modelling

#### Representativeness: Secondary data, time, space are representative

ES assessments can assume temporal, spatial and context-independent representativeness through extrapolating context-, time- and site-specific data. Assessments can rely on secondary data sources (e.g. satellite-derived land cover data), thereby making the assumption that secondary data fit the study purpose although time period of data sources may differ. Snapshot analyses may miss important dynamics in ES provision over time, e.g. single years or seasons (Oteros-Rozas et al. 2014). The rationale behind such assumptions is that ES assessments need to be feasible (i.e. data collection, time, funding and measurement constraints).

Similarly, some methodological approaches use values derived from particular cases as model input for a different study context for the assessment of ES (e.g. Martínez-Harms et al. 2016). These approaches include benefit transfer (transferring ES value data from a study site to another location) and look-up tables (assigning biophysical ES data from, e.g. literature reviews to land cover). This assumes that a specific land use/cover type homogeneously provides a certain amount of ES irrespective of environmental heterogeneity or restricted accessibility and can thus lead to over- or underestimating actual ES provision. Through this approach, linear relationships between land surface and ES provision (i.e. no threshold or saturation effects) are assumed while trade-offs and synergies between ES may actually be expected to vary through spatial scales (Lee and Lautenbach 2016). Benefit transfer also takes average monetary values from published studies and transfers these to other socio-economic contexts. This implicitly assumes that values are transferable and generalizable across socio-economic and cultural contexts and ignores that ES valuations are strongly shaped by socio-cultural context and people’s perceptions (Cáceres et al. 2015).

Average, homogeneous distribution of ES values across large spatial units, for instance recreational values of forests across municipalities (Raudsepp-Hearne et al. 2010), reduces the applicability for conservation problems as it does not allow to detect particularly valuable ecosystems with high conservation priority. Moreover, the credibility and robustness of a transfer of average values to other settings is limited to regions with similar physical landscapes and homogenous social cultures and preferences (Brown et al. 2016).

#### Interactions: ES are independent entities

ES interact and are spatially correlated, resulting in clusters or bundles (Raudsepp-Hearne et al. 2010). ES assessments can analyse interactions between ES to find out whether increasing one ES affects the delivery of another ES in a positive or negative manner (Hölting et al. 2019). The assumption made by these studies is that they can actually identify and characterise relevant interactions. It has been suggested that the direction of the interaction (resulting in trade-offs, synergies or no-effect relationships) depends on the method used to analyse the interactions (Lee and Lautenbach 2016). The choice of thresholds that define directions of ES interactions as either positive, negative or as no effect, for example, strongly influences results. More trade-offs are generally identified through the use of descriptive methods, and it is less likely to identify ‘no-effect’ relationships when using multivariate statistics (Lee and Lautenbach 2016).

Moreover, important synergies and trade-offs may not be detected if the effects of ecosystem functions or intermediate ES on final ES are ignored. For example, trade-offs between timber harvesting, recreation and carbon sequestration fail to be detected when only a potential provision of timber is considered as opposed to studying effects of actual timber harvest on other ES (cf. assumption 4). When important interactions between ES (and biodiversity) are ignored this might lead to wrong decisions in conservation management. Identifying no-effect relationships between provisioning and cultural services where trade-offs actually exist (as discussed for timber production and recreation potential in Lee and Lautenbach 2016) could, for example, lead to insufficient conservation activities, increased timber harvesting and a reduced recreational potential of forests. A better understanding of the underlying thresholds, relationships and interactions can help maximising synergies and minimising undesired trade-offs (Lee and Lautenbach 2016).

#### Expert judgement: Estimation of ES quantities is appropriate

ES assessments employ expert-based judgments to select and assess the quantity of an ES or to weigh the importance of different ES in multi-criteria analyses. An example is the matrix approach (Jacobs et al. 2015), in which land use and land cover are assigned fixed, scaled values from low to high to land cover/use types in order to assess the quantity of provided or used ES (e.g. from 0 to 5). While these approaches provide a feasible way to quantify ES, they assume that ES can be accurately assessed by experts (Cáceres et al. 2015), i.e. scientists or representatives of a stakeholder group. However, as these expert groups may lack social representativeness and include scientists conducting a study, expert-based assessments involving valuations may misjudge preferences of actual users (Campagne et al. 2017), limiting the overall credibility of such approaches. Note that any expert elicitation can have inherent biases, while expert participation in ES assessments might help integrating knowledge on ES in planning and decision-making (Frantzeskaki and Kabisch 2016) or help to close data gaps where field measurements are not feasible (Martin et al. 2012). If ES are assessed by a group that does not include conservation experts or experts who are actively involved in the management of the ecosystem or landscape at stake, important conservation aspects (e.g. target species) might be overlooked (Austin et al. 2016). Expert judgements hence need to include a conservation point of view and need to be validated with place-based local knowledge and field data where possible.

#### Validity: ES indicators are credible

Assumptions also concern the validity of ES indicators as being representative for ES (van Oudenhoven et al. 2018). This includes the use of proxies, e.g. biomass production for provisioning services, species richness for regulating services such as pest control, or artefacts such as hiking trails or viewpoints for cultural services. A consequence of making this assumption is that it becomes unclear what component of ES is actually measured (e.g. a potential or an actual ES, cf. assumption 4) and that comparisons across ES assessments might be misleading.

The choice of ES indicators is crucial for ES assessments that aim to inform conservation decisions. For instance, assessing recreational hiking through the indicator of presence of forest which does not distinguish between old growth and undisturbed or protected parts of the forest (e.g. Raudsepp-Hearne et al. 2010) may ignore different levels of appreciation by people for these different parts of the forest. ES assessments hence need to be accompanied by assessments of conservation relevant aspects of biodiversity and complex ecological relations.

### Socio-economic valuation and value aggregation

#### Economic rationality: People maximise individual utility and have well-informed preferences

Socio-economic ES valuation, i.e. measuring the importance of ES to different groups, relies on concepts and methods from the social sciences (e.g. Wegner and Pascual 2011). Economic valuation studies that rely on revealed or stated preferences typically assume that people are economically rational (see, e.g. Vatn 2009). Economic rationality can imply different aspects, including that people maximise their individual ‘utility’ (an economic notion that reflects a person’s satisfaction), have well-informed preferences about complex and unfamiliar “goods” such as ES (e.g. about underlying ecological functions and the dependence on these ES), that they can articulate their preferences, and that their preferences are stable over time and independent of the social context and setting. Moreover, stated preferences elicit only intended and not actual behaviour and thus, they may only be proxies for actual behaviour (Hausman 2012).

Stated preferences tend to be insensitive to critical aspects such as extent of an ecosystem or the number of species to be valued (Hsee and Rottenstreich 2004). Bartkowski (2017) argues that biodiversity poses challenges for economic valuation as it is not “consumed” like other goods and thus valuing biodiversity cannot rely on pre-defined preferences. Additionally, preferences for species protection may be based on ethical motives or social norms rather than economic rationality. People might actually refuse to state any monetary value if they believe species have an “absolute right to be protected” (Spash et al. 2009, p. 956).

#### Monetary valuation: References can be approximated through monetary measures

Standard economic valuation methods assume that preferences about ES can be approximated through monetary measures (e.g. via stated or revealed willingness to pay). As a consequence, the socio-cultural and psychological complexity behind people’s values, attitudes, motivations, and behaviour is simplified. A wide array of psychological research addresses the complex conceptual and empirical basis between these different notions (Steg et al. 2014) which is usually not addressed in valuation studies. For instance, it has been shown that willingness-to-pay statements are often sensitive to the elicitation method (Lienhoop and Völker 2016). A number of alternative metrics for the valuation of ES that do not rely on monetary metrics have been proposed such as willingness-to-give-up-time or Likert-scale ratings for happiness, life satisfaction, or importance (see Arias-Arévalo et al. 2018 for an overview of methods). Existing monetary approaches can also be combined with deliberative processes, resulting in new methods such as deliberative (democratic) monetary valuation (Orchard-Webb et al. 2016). These approaches intend to consider value plurality but also attempt to inform preferences. Yet, the theoretical foundation of these methods needs to be strengthened (Bartkowski and Lienhoop 2018; Massenberg 2019).

Economic valuation of ES and biodiversity aims to reconcile conservation efforts and development by making important benefits visible. Economic valuation of complex environmental goods should be understood as “imperfect information tool” (Bartkowski and Lienhoop 2018, p. 102). For instance, willingness-to-pay may not capture the full social importance of conserving biodiversity in case of low ability-to-pay and not all values related to biodiversity and ecosystems may be expressed in monetary terms, which implies an incorrect or undervaluation of biodiversity.

#### Aggregation: Summing up welfare across individuals, ES and time

ES valuation may involve several kinds of value aggregations, each of which rely on a number of assumptions. One kind is the aggregation of ES values elicited from individuals or from groups to a representative single value. Different aggregation rules exist (e.g. consensus or majority vote to determine a value among a group), and commonly the mean of all individual values is used. The latter disregards distribution of values across different social groups and may neglect minority voices. Research has shown that different procedures and rules for eliciting values from groups lead to significantly different valuation outcomes (Kenter 2016). Another kind of aggregation combines monetary values across various ES. However, the comparability and substitutability between value dimensions of different ES have been questioned (Spash and Aslaksen 2015). For instance, protecting a sacred landscape cannot be compared to—or substituted by—the exploitation of a provisioning service. A third kind of aggregation is done for ES benefits and costs that accrue over longer periods of time. Social discounting is typically used to weigh the social benefits and costs of ES at different time periods and to reduce it to a single figure (“net present value”). This approach allows to compare the desirability of different future trajectories. Any social discount rate, however, is the result of numerous assumptions regarding intergenerational justice, substitutability concerns, or projections of future welfare and economic growth (Gowdy et al. 2010).

Aggregation of values across ES hides who is benefiting and losing from their provision. For instance, the value in monetary terms that local communities derive from a forest may be negligible compared to the values of global commons such as a stable climate or biodiversity. Yet, these values can be crucial from justice considerations, which need to be considered in conservation policy. With respect to value aggregation over time, the typical pattern is a scenario with more intensive use of ES (e.g. deforestation for timber and agriculture, intensive fishing) that leads to higher values in the short term, whereas conservation leads to a more stable value flow with eventually higher ES provision in the longer term. The choice of the social discount rate can fundamentally shift conclusions as to which scenario is preferable from a societal perspective.

### Using results for decision-making

#### Relevance: ES are important for conservation decisions

ES assessments can assume relevance for societal decisions that affect conservation (Posner et al. 2016). Information from assessments is assumed to raise awareness, and to lead to more informed decision-making and action (Blicharska and Hilding-Rydevik 2018). McKenzie et al. (2014) have distinguished three levels of potential use of ES knowledge in decision-making: (i) conceptual use, i.e. generating a principle understanding of how ecosystems support well-being; (ii) strategic use, i.e. supporting or justifying interventions or beliefs; (iii) instrumental use, i.e. information is directly used in decisions. Evidence suggests that ES knowledge is used at the conceptual level in stakeholder interactions, but that it is rarely directly influencing decision-making related to conservation (Saarikoski et al. 2018). Valuing ES can involve advocacy towards particular conservation decisions (Laurans and Mermet 2014). This strategic rationale seems especially pronounced when ES valuations highlight large economic values in monetary units as an argument to support conservation.

The relevance of ES assessments and in particular valuations in decision-making remains unclear (e.g. Laurans et al. 2013). Moreover, it remains unclear whether ES value information is effective to support decisions in favour of conservation (Rode et al. 2017). Decision-makers appreciate a plurality of information, and even explicitly request to include non-monetary next to monetary information about ES (Ruckelshaus et al. 2015).

## Discussion and conclusion: How to deal with assumptions?

Assumptions are necessarily being made in ES assessments. These include implicitly or explicitly made assumptions related to conceptual and ethical foundations, to data collection, indication, mapping, and modelling, to socio-economic valuation and value aggregation, as well as to using results for decision-making.

Assumptions are not a bad thing per se (Evans 2019). Pragmatism can play a major role when conducting ES assessments in accordance with available resources (e.g. time, data, knowledge; van Oudenhoven et al. 2018). ES assessments are often set in a mission-driven arena that strives to inform policy to conserve biodiversity quickly and hence pragmatic methodological and conceptual choices are made which may simplify systems through making assumptions. In the same line, a subtle balance between referring to a common language and acknowledging context-specific conditions of human–nature relationships needs to be found in each project if scientific results are to be relevant to policy and practice (Díaz et al. 2018).

However, as ES science is both built and used in inter- or transdisciplinary contexts, assumptions underlying one’s own discipline may not be clear to others. The adoption of methods from other fields may lead to unintended and hidden assumptions. In particular, knowledge involving uncertainties that are not made explicit might be (mis-)interpreted as a supposedly certain outcome. This could then lead to wrong interpretations or adverse recommendations for conservation policies (Fig. 1) and for policies which are interacting with or related to conservation. Thus, we advocate for an increased attention to transparency about assumptions in ES assessments, in particular through clarifying their potential impacts on study outcomes for conservation, e.g. through using standardised templates based on the typology developed here, to assess whether assumptions are used in a study and how they might influence study implementation and outcome. At the same time, tests and validations of important assumptions need to be put forward more strongly in the ES research agenda in order to close knowledge gaps. This could be done through systematically testing, e.g. the prevalence of worldviews among stakeholders (assumption 1), the acceptance of terminology (3), tests of uncertainties with field-collected data (5, 6, 7), tests of the effect of using different indicators (8) or of aggregation procedures (11), and empirical assessments on actual post-study uptake of ES assessments in policy (12). Finally, conservation scientists, practitioners and decision-makers should adopt strategies to deal with assumptions. Here, we provide a comprehensive list of potential strategies (see Table 1). Overall they rely on an increased attention and time-dedication to making definitions and values at stake explicit, as well as shedding light on what is included and what is (intentionally) ignored in the assessment, both conceptually and methodologically.

## Electronic supplementary material

Below is the link to the electronic supplementary material.

Supplementary file1 (PDF 205 kb)
