# Supplementary material for: Assumptions in ecosystem service assessments: Increasing transparency for conservation
Source: Ambio. 2020 Sep 11;50(2):289–300. doi: 10.1007/s13280-020-01379-9 (PMC7782657; doi:10.1007/s13280-020-01379-9)
Supplement: Supplementary file 1 — Supplementary file1 (PDF 205 kb) [file 13280_2020_1379_MOESM1_ESM.pdf]

Ambio
Electronic Supplementary Material
This supplementary material has not been peer reviewed

Title: **Assumptions in ecosystem service assessments: Increasing transparency for conservation**

Matthias Schröter, Emilie Crouzat, Lisanne Hölting, Julian Massenberg, Julian Rode, Mario Hanisch, Nadja Kabisch, Julia Palliwoda, Jörg A. Priess, Ralf Seppelt, Michael Beckmann

Table S1: Participants of the workshop

Participants (anonymised)	Career stage	Discipline
Participant 1	Postdoc	Environmental sciences
Participant 2	Postdoc	Terrestrial ecology
Participant 3	Postdoc	Environmental sciences
Participant 4	PhD candidate	Environmental sciences
Participant 5	PhD candidate	Environmental/ecological economics
Participant 6	Senior scientist	Behavioural economics
Participant 7	PhD candidate	Geo-ecology
Participant 8	Senior scientist	Urban ecology
Participant 9	PhD candidate	Urban ecology
Participant 10	Senior scientist	Environmental sciences
Participant 11	Professor	Environmental sciences
Participant 12	Senior scientist	Environmental/ecological economics
Participant 13	Senior scientist	Environmental sciences
Participant 14	Professor	Conservation biology

Table S2: Extraction sheet used for review of the papers, based on the preliminary list of assumptions identified during the expert workshop

**A) Using the ecosystem service concept**	**1. Implicit underlying philosophical assumptions**
	Consequentialism, anthropocentrism, instrumentalism
	Nature is (only) positive
	Multifunctionality is positive/sustainable
	Level of ES use is sustainable
	ES concept applicable globally
	**2. Assuming that different aspects/components of ecosystem services are conceptually equal**
	Ecological processes, potential provision, use, demand are set equal (indicator and conceptual level)
**B) Biophysical data collection, indication, mapping, and modelling**	**3. Assuming temporal, spatial and context-specific appropriateness and representativity for different study purposes.**
	Appropriateness of 1-time step analysis
	Appropriateness of homogeneous spatial distribution
	Closed systems, provision of ES across scales neglected
	Representativity or appropriateness of ES lists
	**4. Assuming reliability of ecosystem service indicators.**
	Indicators credibly represent different aspects of ES
	**5. Assuming temporal and spatial stationarity.**
	Temporal dynamics constant, state is not close to ecological thresholds
	Underlying social-ecological conditions stable, demand stable over time
	Land use and land cover as ES indicators
	**6. Assuming quantities and values of ecosystem services through expert-based assessments.**
	Expert-based judgements
	Bayesian belief networks (weighing and interactions)
	Who is an expert and how different knowledge systems may agree or disagree
	**7. Assuming no interactions between ecosystem services.**
	Compatibility: provision of one ES does not affect the provision of another ES
	Optimisation for only one ES while ignoring interactions.
**C) Socio-economic valuation and value aggregation**	**8. Assuming that people are economically rational**
	Neo-classical economic theory: economic rationality, maximize individual utility, have complete information, know their preferences, have preferences that are stable over time and independent of social context
	Utility can be approximated through monetary measures
	**9. Assuming transferability and generalisability of values across socioeconomic and cultural contexts.**
	ES values independent of the socio-economic and cultural context
	Benefit transfer of average value coefficients: homogeneous distribution, comparable socio-economic context, and similarity across time
	**10. Assuming that welfare can be aggregated over space and time.**
	Aggregating individual values meaningful
	Equal importance of multiple ES
	Two values from different value domains can be compared and traded-off
**D) Using results for decision-making**	**11. Assuming relevance for societal decisions.**
	Selection and a quantification of assessed ES is representative for a concerned group of stakeholders
	Raises awareness, is policy relevant and applicable, and motivates decision-makers to take action.
	Strategic use for environmental advocacy, in particular monetary valuation relevant for decision making

Table S3: 70 published ecosystem service assessments that were used to refine the typology.

Authors	Year	Title	Journal	DOI
Raudsepp-Hearne, C; Peterson, GD; Bennett, EM	2010	Ecosystem service bundles for analyzing tradeoffs in diverse landscapes	PROCEEDINGS OF THE NATIONAL ACADEMY OF SCIENCES OF THE UNITED STATES OF AMERICA	10.1073/pnas.0907284107
Eigenbrod, F; Armsworth, PR; Anderson, BJ; Heinemeyer, A; Gillings, S; Roy, DB; Thomas, CD; Gaston, KJ	2010	The impact of proxy-based methods on mapping the distribution of ecosystem services	JOURNAL OF APPLIED ECOLOGY	10.1111/j.1365-2664.2010.01777.x
Bryan, BA; Raymond, CM; Crossman, ND; Macdonald, DH	2010	Targeting the management of ecosystem services based on social values: Where, what, and how?	LANDSCAPE AND URBAN PLANNING	10.1016/j.landurbplan.2010.05.002
Birch, JC; Newton, AC; Aquino, CA; Cantarello, E; Echeverria, C; Kitzberger, T; Schiappacasse, I; Garavito, NT	2010	Cost-effectiveness of dryland forest restoration evaluated by spatial analysis of ecosystem services	PROCEEDINGS OF THE NATIONAL ACADEMY OF SCIENCES OF THE UNITED STATES OF AMERICA	10.1073/pnas.1003369107
Li, TH; Li, WK; Qian, ZH	2010	Variations in ecosystem service value in response to land use changes in Shenzhen	ECOLOGICAL ECONOMICS	10.1016/j.ecolecon.2008.05.018
Jenkins, WA; Murray, BC; Kramer, RA; Faulkner, SP	2010	Valuing ecosystem services from wetlands restoration in the Mississippi Alluvial Valley	ECOLOGICAL ECONOMICS	10.1016/j.ecolecon.2009.11.022
de Lange, WJ; van Wilgen, BW	2010	An economic assessment of the contribution of biological control to the management of invasive alien plants and to the protection of ecosystem services in South Africa	BIOLOGICAL INVASIONS	10.1007/s10530-010-9811-y
Liu, SA; Costanza, R; Troy, A; D'Aagostino, J; Mates, W	2010	Valuing New Jersey's Ecosystem Services and Natural Capital: A Spatially Explicit Benefit Transfer Approach	ENVIRONMENTAL MANAGEMENT	10.1007/s00267-010-9483-5
Eigenbrod, F; Armsworth, PR; Anderson, BJ; Heinemeyer, A; Gillings, S; Roy, DB; Thomas, CD; Gaston, KJ	2010	Error propagation associated with benefits transfer-based mapping of ecosystem services	BIOLOGICAL CONSERVATION	10.1016/j.biocon.2010.06.015
Brenner, J; Jimenez, JA; Sarda, R; Garola, A	2010	An assessment of the non-market value of the ecosystem services provided by the Catalan coastal zone, Spain	OCEAN & COASTAL MANAGEMENT	10.1016/j.ocecoaman.2009.10.008
Sherrouse, BC; Clement, JM;	2011	A GIS application for assessing, mapping, and quantifying	APPLIED GEOGRAPHY	10.1016/j.apgeog.2010.08.

Semmens, DJ		the social values of ecosystem services		002
Davies, ZG; Edmondson, JL; Heinemeyer, A; Leake, JR; Gaston, KJ	2011	Mapping an urban ecosystem service: quantifying above-ground carbon storage at a city-wide scale	JOURNAL OF APPLIED ECOLOGY	10.1111/j.1365-2664.2011.02021.x
Dobbs, C; Escobedo, FJ; Zipperer, WC	2011	A framework for developing urban forest ecosystem services and goods indicators	LANDSCAPE AND URBAN PLANNING	10.1016/j.landurbplan.2010.11.004
Lautenbach, S; Kugel, C; Lausch, A; Seppelt, R	2011	Analysis of historic changes in regional ecosystem service provisioning using land use data	ECOLOGICAL INDICATORS	10.1016/j.ecolind.2010.09.007
Swetnam, RD; Fisher, B; Mbilinyi, BP; Munishi, PKT; Willcock, S; Ricketts, T; Mwakalila, S; Balmford, A; Burgess, ND; Marshall, AR; Lewis, SL	2011	Mapping socio-economic scenarios of land cover change: A GIS method to enable ecosystem service modelling	JOURNAL OF ENVIRONMENTAL MANAGEMENT	10.1016/j.jenvman.2010.09.007
Kenter, JO; Hyde, T; Christie, M; Fazey, I	2011	The importance of deliberation in valuing ecosystem services in developing countries-Evidence from the Solomon Islands	GLOBAL ENVIRONMENTAL CHANGE-HUMAN AND POLICY DIMENSIONS	10.1016/j.gloenvcha.2011.01.001
Martin-Lopez, B; Garcia-Llorente, M; Palomo, I; Montes, C	2011	The conservation against development paradigm in protected areas: Valuation of ecosystem services in the Donana social-ecological system (southwestern Spain)	ECOLOGICAL ECONOMICS	10.1016/j.ecolecon.2011.03.009
Higgins, CB; Stephenson, K; Brown, BL	2011	Nutrient Bioassimilation Capacity of Aquacultured Oysters: Quantification of an Ecosystem Service	JOURNAL OF ENVIRONMENTAL QUALITY	10.2134/jeq2010.0203
Watanabe, MDB; Ortega, E	2011	Ecosystem services and biogeochemical cycles on a global scale: valuation of water, carbon and nitrogen processes	ENVIRONMENTAL SCIENCE & POLICY	10.1016/j.envsci.2011.05.013
Fisher, B; Turner, RK; Burgess, ND; Swetnam, RD; Green, J; Green, RE; Kajembe, G; Kulindwa, K; Lewis, SL; Marchant, R; Marshall, AR; Madoffe, S; Munishi, PKT; Morse-Jones, S; Mwakalila, S; Paavola, J; Naidoo, R; Ricketts, T; Rouget, M; Willcock, S; White, S; Balmford, A	2011	Measuring, modeling and mapping ecosystem services in the Eastern Arc Mountains of Tanzania	PROGRESS IN PHYSICAL GEOGRAPHY	10.1177/0309133311422968
van Oudenhoven, APE; Petz, K; Alkemade, R; Hein, L; de Groot, RS	2012	Framework for systematic indicator selection to assess effects of land management on ecosystem services	ECOLOGICAL INDICATORS	10.1016/j.ecolind.2012.01.012

White, C; Halpern, BS; Kappel, CV	2012	Ecosystem service tradeoff analysis reveals the value of marine spatial planning for multiple ocean uses	PROCEEDINGS OF THE NATIONAL ACADEMY OF SCIENCES OF THE UNITED STATES OF AMERICA	10.1073/pnas.1114215109
Koschke, L; Furst, C; Frank, S; Makeschin, F	2012	A multi-criteria approach for an integrated land-cover-based assessment of ecosystem services provision to support landscape planning	ECOLOGICAL INDICATORS	10.1016/j.ecolind.2011.12.010
Lu, YH; Fu, BJ; Feng, XM; Zeng, Y; Liu, Y; Chang, RY; Sun, G; Wu, BF	2012	A Policy-Driven Large Scale Ecological Restoration: Quantifying Ecosystem Services Changes in the Loess Plateau of China	PLOS ONE	10.1371/journal.pone.0031782
Haines-Young, R; Potschin, M; Kienast, F	2012	Indicators of ecosystem service potential at European scales: Mapping marginal changes and trade-offs	ECOLOGICAL INDICATORS	10.1016/j.ecolind.2011.09.004
Kroll, F; Muller, F; Haase, D; Fohrer, N	2012	Rural-urban gradient analysis of ecosystem services supply and demand dynamics	LAND USE POLICY	10.1016/j.landusepol.2011.07.008
Su, SL; Xiao, R; Jiang, ZL; Zhang, Y	2012	Characterizing landscape pattern and ecosystem service value changes for urbanization impacts at an eco-regional scale	APPLIED GEOGRAPHY	10.1016/j.apgeog.2011.12.001
Grabowski, JH; Brumbaugh, RD; Conrad, RF; Keeler, AG; Opaluch, JJ; Peterson, CH; Piehler, MF; Powers, SP; Smyth, AR	2012	Economic Valuation of Ecosystem Services Provided by Oyster Reefs	BIOSCIENCE	10.1525/bio.2012.62.10.10
Tengberg, A; Fredholm, S; Eliasson, I; Knez, I; Saltzman, K; Wetterberg, O	2012	Cultural ecosystem services provided by landscapes: Assessment of heritage values and identity	ECOSYSTEM SERVICES	10.1016/j.ecoser.2012.07.006
Nedkov, S; Burkhard, B	2012	Flood regulating ecosystem services-Mapping supply and demand, in the Etropole municipality, Bulgaria	ECOLOGICAL INDICATORS	10.1016/j.ecolind.2011.06.022
Allan, JD; McIntyre, PB; Smith, SDP; Halpern, BS; Boyer, GL; Buchsbaum, A; Burton, GA; Campbell, LM; Chadderton, WL; Ciborowski, JJH; Doran, PJ; Eder, T; Infante, DM; Johnson, LB; Joseph, CA; Marino, AL; Prusevich, A; Read, JG; Rose, JB; Rutherford, ES; Sowa, SP; Steinman,	2013	Joint analysis of stressors and ecosystem services to enhance restoration effectiveness	PROCEEDINGS OF THE NATIONAL ACADEMY OF SCIENCES OF THE UNITED STATES OF AMERICA	10.1073/pnas.1213841110

AD				
Palomo, I; Martin-Lopez, B; Potschin, M; Haines-Young, R; Montes, C	2013	National Parks, buffer zones and surrounding lands: Mapping ecosystem service flows	ECOSYSTEM SERVICES	10.1016/j.ecoser.2012.09.001
Larondelle, N; Haase, D	2013	Urban ecosystem services assessment along a rural-urban gradient: A cross-analysis of European cities	ECOLOGICAL INDICATORS	10.1016/j.ecolind.2012.12.022
Feng, XM; Fu, BJ; Lu, N; Zeng, Y; Wu, BF	2013	How ecological restoration alters ecosystem services: an analysis of carbon sequestration in China's Loess Plateau	SCIENTIFIC REPORTS	10.1038/srep02846
Leh, MDK; Matlock, MD; Cummings, EC; Nalley, LL	2013	Quantifying and mapping multiple ecosystem services change in West Africa	AGRICULTURE ECOSYSTEMS & ENVIRONMENT	10.1016/j.agee.2012.12.001
Garcia-Nieto, AP; Garcia-Llorente, M; Iniesta-Arandia, I; Martin-Lopez, B	2013	Mapping forest ecosystem services: From providing units to beneficiaries	ECOSYSTEM SERVICES	10.1016/j.ecoser.2013.03.003
Jackson, B; Pagella, T; Sinclair, F; Orellana, B; Henshaw, A; Reynolds, B; Mcintyre, N; Wheater, H; Eycott, A	2013	Polyscape: A GIS mapping framework providing efficient and spatially explicit landscape-scale valuation of multiple ecosystem services	LANDSCAPE AND URBAN PLANNING	10.1016/j.landurbplan.2012.12.014
Butterfield, BJ; Suding, KN	2013	Single-trait functional indices outperform multi-trait indices in linking environmental gradients and ecosystem services in a complex landscape	JOURNAL OF ECOLOGY	10.1111/1365-2745.12013
Butler, JRA; Wong, GY; Metcalfe, DJ; Honzak, M; Pert, PL; Rao, N; van Grieken, ME; Lawson, T; Bruce, C; Kroon, FJ; Brodie, JE	2013	An analysis of trade-offs between multiple ecosystem services and stakeholders linked to land use and water quality management in the Great Barrier Reef, Australia	AGRICULTURE ECOSYSTEMS & ENVIRONMENT	10.1016/j.agee.2011.08.017
Fontana, V; Radtke, A; Fedrigotti, VB; Tappeiner, U; Tasser, E; Zerbe, S; Buchholz, T	2013	Comparing land-use alternatives: Using the ecosystem services concept to define a multi-criteria decision analysis	ECOLOGICAL ECONOMICS	10.1016/j.ecolecon.2013.05.007
van Berkel, DB; Verburg, PH	2014	Spatial quantification and valuation of cultural ecosystem services in an agricultural landscape	ECOLOGICAL INDICATORS	10.1016/j.ecolind.2012.06.025
Schroter, M; Barton, DN; Remme, RP; Hein, L	2014	Accounting for capacity and flow of ecosystem services: A conceptual model and a case study for Telemark, Norway	ECOLOGICAL INDICATORS	10.1016/j.ecolind.2013.09.018
Paracchini, ML; Zulian, G; Kopperoinen, L; Maes, J; Schagner, JP; Termansen, M; Zandersen, M; Perez-Soba, M; Scholefield, PA;	2014	Mapping cultural ecosystem services: A framework to assess the potential for outdoor recreation across the EU	ECOLOGICAL INDICATORS	10.1016/j.ecolind.2014.04.018

Bidoglio, G				
Schulp, CJE; Lautenbach, S; Verburg, PH	2014	Quantifying and mapping ecosystem services: Demand and supply of pollination in the European Union	ECOLOGICAL INDICATORS	10.1016/j.ecolind.2013.07.014
Bagstad, KJ; Villa, F; Batker, D; Harrison-Cox, J; Voigt, B; Johnson, GW	2014	From theoretical to actual ecosystem services: mapping beneficiaries and spatial flows in ecosystem service assessments	ECOLOGY AND SOCIETY	10.5751/ES-06523-190264
Serna-Chavez, HM; Schulp, CJE; van Bodegom, PM; Bouten, W; Verburg, PH; Davidson, MD	2014	A quantitative framework for assessing spatial flows of ecosystem services	ECOLOGICAL INDICATORS	10.1016/j.ecolind.2013.11.024
Sturck, J; Poortinga, A; Verburg, PH	2014	Mapping ecosystem services: The supply and demand of flood regulation services in Europe	ECOLOGICAL INDICATORS	10.1016/j.ecolind.2013.11.010
Larondelle, N; Haase, D; Kabisch, N	2014	Mapping the diversity of regulating ecosystem services in European cities	GLOBAL ENVIRONMENTAL CHANGE-HUMAN AND POLICY DIMENSIONS	10.1016/j.gloenvcha.2014.04.008
Oteros-Rozas, E; Martin-Lopez, B; Gonzalez, JA; Plieninger, T; Lopez, CA; Montes, C	2014	Socio-cultural valuation of ecosystem services in a transhumance social-ecological network	REGIONAL ENVIRONMENTAL CHANGE	10.1007/s10113-013-0571-y
Iniesta-Arandia, I; Garcia-Llorente, M; Aguilera, PA; Montes, C; Martin-Lopez, B	2014	Socio-cultural valuation of ecosystem services: uncovering the links between values, drivers of change, and human well-being	ECOLOGICAL ECONOMICS	10.1016/j.ecolecon.2014.09.028
Queiroz, C; Meacham, M; Richter, K; Norstrom, AV; Andersson, E; Norberg, J; Peterson, G	2015	Mapping bundles of ecosystem services reveals distinct types of multifunctionality within a Swedish landscape	AMBIO	10.1007/s13280-014-0601-0
Song, W; Deng, XZ; Yuan, YW; Wang, Z; Li, ZH	2015	Impacts of land-use change on valued ecosystem service in rapidly urbanized North China Plain	ECOLOGICAL MODELLING	10.1016/j.ecolmodel.2015.01.029
Derkzen, ML; van Teeffelen, AJA; Verburg, PH	2015	REVIEW Quantifying urban ecosystem services based on high-resolution data of urban green space: an assessment for Rotterdam, the Netherlands	JOURNAL OF APPLIED ECOLOGY	10.1111/1365-2664.12469
Baro, F; Haase, D; Gomez-Baggethun, E; Frantzeskaki, N	2015	Mismatches between ecosystem services supply and demand in urban areas: A quantitative assessment in five European cities	ECOLOGICAL INDICATORS	10.1016/j.ecolind.2015.03.013
Richards, DR; Friess, DA	2015	A rapid indicator of cultural ecosystem service usage at a	ECOLOGICAL INDICATORS	10.1016/j.ecolind.2015.01.

		fine spatial scale: Content analysis of social media photographs		034
Hayha, T; Franzese, PP; Paletto, A; Fath, BD	2015	Assessing, valuing, and mapping ecosystem services in Alpine forests	ECOSYSTEM SERVICES	10.1016/j.ecoser.2015.03.001
Blaen, PJ; Jia, L; Peh, KSH; Field, RH; Balmford, A; MacDonald, MA; Bradbury, RB	2015	Rapid Assessment of Ecosystem Services Provided by Two Mineral Extraction Sites Restored for Nature Conservation in an Agricultural Landscape in Eastern England	PLOS ONE	10.1371/journal.pone.0121010
Garcia-Nieto, AP; Quintas-Soriano, C; Garcia-Llorente, M; Palomo, I; Montes, C; Martin-Lopez, B	2015	Collaborative mapping of ecosystem services: The role of stakeholders' profiles	ECOSYSTEM SERVICES	10.1016/j.ecoser.2014.11.006
Liquete, C; Kleeschulte, S; Dige, G; Maes, J; Grizzetti, B; Olah, B; Zulian, G	2015	Mapping green infrastructure based on ecosystem services and ecological networks: A Pan-European case study	ENVIRONMENTAL SCIENCE & POLICY	10.1016/j.envsci.2015.07.009
Crouzat, E; Mouchet, M; Turkelboom, F; Byczek, C; Meersmans, J; Berger, F; Verkerk, PJ; Lavorel, S	2015	Assessing bundles of ecosystem services from regional to landscape scale: insights from the French Alps	JOURNAL OF APPLIED ECOLOGY	10.1111/1365-2664.12502
Tenerelli, P; Demsar, U; Luque, S	2016	Crowdsourcing indicators for cultural ecosystem services: A geographically weighted approach for mountain landscapes	ECOLOGICAL INDICATORS	10.1016/j.ecolind.2015.12.042
Kindu, M; Schneider, T; Teketay, D; Knoke, T	2016	Changes of ecosystem service values in response to land use/land cover dynamics in Munessa-Shashemene landscape of the Ethiopian highlands	SCIENCE OF THE TOTAL ENVIRONMENT	10.1016/j.scitotenv.2015.12.127
Czembrowski, P; Kronenberg, J	2016	Hedonic pricing and different urban green space types and sizes: Insights into the discussion on valuing ecosystem services	LANDSCAPE AND URBAN PLANNING	10.1016/j.landurbplan.2015.10.005
Tayyebi, A; Meehan, TD; Dischler, J; Radloff, G; Ferris, M; Gratton, C	2016	SmartScape (TM): A web-based decision support system for assessing the tradeoffs among multiple ecosystem services under crop-change scenarios	COMPUTERS AND ELECTRONICS IN AGRICULTURE	10.1016/j.compag.2015.12.003
Baro, F; Palomo, I; Zulian, G; Vizcaino, P; Haase, D; Gomez-Baggethun, E	2016	Mapping ecosystem service capacity, flow and demand for landscape and urban planning: A case study in the Barcelona metropolitan region	LAND USE POLICY	10.1016/j.landusepol.2016.06.006

Kremer, P; Hamstead, ZA; McPhearson, T	2016	The value of urban ecosystem services in New York City: A spatially explicit multicriteria analysis of landscape scale valuation scenarios	ENVIRONMENTAL SCIENCE & POLICY	10.1016/j.envsci.2016.04.012
Jiang, C; Li, DQ; Wang, DW; Zhang, LB	2016	Quantification and assessment of changes in ecosystem service in the Three-River Headwaters Region, China as a result of climate variability and land cover change	ECOLOGICAL INDICATORS	10.1016/j.ecolind.2016.01.051
Boithias, L; Terrado, M; Corominas, L; Ziv, G; Kumar, V; Marques, M; Schuhmacher, M; Acuna, V	2016	Analysis of the uncertainty in the monetary valuation of ecosystem services - A case study at the river basin scale	SCIENCE OF THE TOTAL ENVIRONMENT	10.1016/j.scitotenv.2015.11.066
Bryce, R; Irvine, KN; Church, A; Fish, R; Ranger, S; Kenter, JO	2016	Subjective well-being indicators for large-scale assessment of cultural ecosystem services	ECOSYSTEM SERVICES	10.1016/j.ecoser.2016.07.015
Albert, C; Galler, C; Hermes, J; Neuendorf, F; von Haaren, C; Lovett, A	2016	Applying ecosystem services indicators in landscape planning and management: The ES-in-Planning framework	ECOLOGICAL INDICATORS	10.1016/j.ecolind.2015.03.029
